# Combinatorial Growth of Vertically Aligned Nanocomposite Thin Films for Accelerated Exploration in Composition Variation

**DOI:** 10.1002/smsc.202300049

**Published:** 2023-09-27

**Authors:** Bethany X. Rutherford, Di Zhang, Lizabeth Quigley, James P. Barnard, Bo Yang, Juanjuan Lu, Sundar Kunwar, Hongyi Dou, Jianan Shen, Aiping Chen, Haiyan Wang

**Affiliations:** ^1^ School of Materials Engineering Purdue University West Lafayette Indiana 47907 USA; ^2^ Center for Integrated Technology (CINT) Los Alamos National Laboratory Los Alamos New Mexico 87545 USA; ^3^ School of Electrical and Computer Engineering Purdue University West Lafayette Indiana 47907 USA

**Keywords:** combinatorial growth, composition, nanocomposite, thin film, vertically aligned nanocomposite

## Abstract

Combinatorial growth is capable of creating a compositional gradient for thin film materials and thus has been adopted to explore composition variation mostly for metallic alloy thin films and some dopant concentrations for ceramic thin films. This study uses a combinatorial pulsed laser deposition method to successfully fabricate two‐phase oxide–oxide vertically aligned nanocomposite (VAN) thin films of La_0.7_Sr_0.3_MnO_3_ (LSMO)‐NiO with variable composition across the film area. The LSMO‐NiO compositional gradient across the film alters the two‐phase morphology of the VAN through varying nanopillar size and density. Additionally, the magnetic anisotropy and magnetoresistance properties of the nanocomposite thin films increase with increasing NiO composition. This demonstration of a combinatorial method for VAN growth can increase the efficiency of nanocomposite thin film research by allowing all possible compositions of thin film materials to be explored in a single deposition.

## Graphical abstract

A combinatorial growth method, as an accelerated approach to explore the composition variation of vertically aligned nanocomposites, using LaSrMnO3:NiO as a model system, is presented. Tunable composition, two‐phase morphology, magnetic properties, and magnetoresistance properties are effectively tuned in one sample, achieved in a single run.

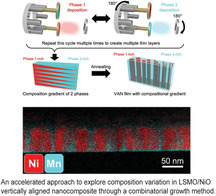

## 1 Introduction

Combinatorial growth methods for thin films have been implemented since the 1990s for expedited material exploration. The technique has previously been used to alloy two or more metals to find the composition yielding the optimal or desired properties of an alloy.^[^ ^1^, ^2^ ^]^ For oxide thin film materials, combinatorial growth has been used to tune the ratio of two elements in an oxide for optimal material properties.^[^ ^3^, ^4^, ^5^, ^6^, ^7^, ^8^, ^9^, ^10^ ^]^ Combinatorial methods have also been used to explore the properties of metal dopants to an oxide material and the effects of oxygen content in VO_2_.^[^ ^11^, ^12^ ^]^ Previous studies have implemented a masking technique with sputtering or molecular beam epitaxy to cover parts of the substrate or thin film to create different quadrants of thin film materials such as Mg_*x*_Zn_1−*x*_O.^[^ ^3^, ^4^ ^]^ Other methods involve implementing sputtering and an understanding of the plume locations for different guns and no substrate rotation such as in the study of the Ni–Mn–Ga shape memory alloy.^[^ ^2^, ^6^, ^12^ ^]^ Up to date, the possibility of implementing combinatorial growth to explore nanocomposite thin film materials and thin films has not yet been demonstrated.

Vertically aligned nanocomposite (VAN) thin films combine two or more phases of materials to create a thin film with a nanostructure where one or more phases become vertical nanopillars and one or more phases form a matrix.^[^ ^13^ ^]^ These materials are created by carefully selected phases that are immiscible and bring about multifunctionality by combining materials with different physical properties. These multifunctional materials have various applications in data storage, memristors, photonic devices, superconductors, and transistors.^[^ ^14^, ^15^, ^16^, ^17^ ^]^ To fabricate these multifunctional VAN thin films, pulsed laser deposition (PLD) is widely used with individual targets with each unique composition prepared for deposition.^[^ ^18^, ^19^, ^20^, ^21^, ^22^ ^]^

There are a few conventional ways to fabricate VAN thin films including composite targets with a premixed phase ratio, multitarget systems where each target is a different phase, and in the case of metal‐oxide VAN systems, a metal strip can be used in conjunction with the oxide target.^[^ ^23^, ^24^ ^]^ VAN composition studies require one to fabricate multiple thin films from the different composite targets or variations of pulses on each of the individual targets. This process is time‐consuming and inefficient due to the many samples needed to test all of the material compositions. A slight modification of the above setup will allow the synthesis of VANs using a combinatorial growth method. This approach can accelerate the VAN thin film research by exploring all possible compositions in one growth. This method reduces many other compositions such as laser energy variation and substrate quality variation. Ross et al. reported the combinatorial synthesis of BiFeO_3_‐CoFe_2_O_4_ multiferroic VANs and studied film strain and magnetization.^[^ ^25^ ^]^ To grow VANs, each layer was kept below one unit cell to limit superlattice formation. In fact, the combinatorial growth of VAN thin films is largely unexplored in terms of design principles and functionality tuning.

In this work, La_0.7_Sr_0.3_MnO_3_ (LSMO)‐NiO VAN was selected as a model system to explore the feasibility of VAN growth via the combinatorial method for efficient composition, microstructure, and functionality modulation. The LSMO‐NiO system was selected for a few reasons. First, LSMO‐NiO nanocomposites were successfully grown in multiple studies.^[^ ^17^, ^25^, ^26^, ^27^, ^28^, ^29^ ^]^ In one of the recent LSMO‐NiO nanocomposites reports,^[^ ^28^ ^]^ two targets of LSMO and NiO were used instead of the single mixed target reported in other articles. These prior LSMO:NiO works all provide a solid foundation for the combinatorial growth demonstration in this work, and allows us to focus more on the innovative aspects for the implementation of a combinatorial growth method, rather than on the basic feasibility of the VAN system itself. Second, considering the physical properties, LSMO is a known perovskite manganite, and well recognized for its colossal magnetoresistance and half‐metallic properties, and has potential applications in spintronic devices.^[^ ^26^ ^]^ NiO, in contrast, is a known antiferromagnetic oxide, with potential applications in magnetic and spintronic devices due to its properties such as high resistivity and large bandgap. By combining these two materials, we aimed to achieve a novel system that could exhibit unique magnetic and electrical transport properties.^[^ ^26^ ^]^ The VAN system of LSMO‐NiO may create interesting functions such as exchange bias coupling and magneto‐transport where the phases experience interfacial coupling.^[^ ^17^, ^30^ ^]^ Moreover, LSMO has a lattice parameter of 3.87 Å, and NiO has a lattice parameter of 4.17 Å, which allows for a large strain coupling between the two phases.

To evaluate the feasibility of this combinatorial growth method, X‐ray diffraction (XRD), transmission electron microscopy (TEM), scanning transmission electron microscopy (STEM), energy‐dispersive X‐ray spectroscopy (EDX), and scanning electron microscopy (SEM) were used to evaluate the nanostructure of different compositions along the gradient. Various sample locations with different film compositions varied from NiO‐rich to LSMO‐rich were characterized for microstructure properties, ferromagnetic, and low‐field magnetoresistance (MR) properties.

## 2 Results and Discussion

**Figure 1:**
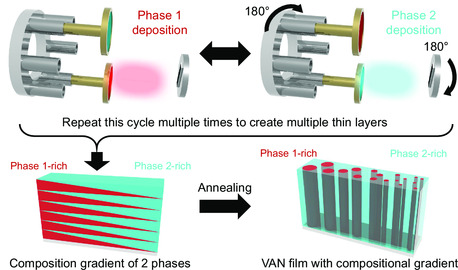
Combinatorial growth process for two‐phase oxide–oxide VAN thin film materials. The layered deposition is fabricated by alternating the targets and substrate material ends to create nanolayers of a few unit cells thick to create the composition gradient between the two phases. The layered structure is then annealed into a VAN structure.

To demonstrate this method, separate targets for each of the selected phases are needed along with a single substrate material that covers the areas where deposition is desired. **Figure** **1** shows the setup and the steps for a two‐phase oxide–oxide nanocomposite growth. Specifically, the center of the plume is aligned with the ends of the substrate material to maximize the amount of phase 1 (NiO) at the end of the composition gradient. After depositing a thin layer of phase 1, the target carousel and substrate holder were rotated 180° to line up the plume center with the other end of the substrate material to create the composition gradient. Phase 2 material (i.e., LSMO) is also deposited in a very thin layer before returning the setup to the initial position. This process is repeated until the desired thickness or number of layers is achieved, and the location of the substrate in relation to the deposition plume determines the ratio of the two materials. A thin film with a composition gradient between the two phases is achieved through the stacking of these layers as seen in the schematic of Figure 1. Each layer is made to be extremely thin (<5 nm) to allow for interdiffusion to occur between the layers during the postannealing process. This postannealing process allows for the formation of the VAN structure at each composition as seen in the final step of Figure 1. This unique combinatorial growth technique could expedite the exploration of VAN thin films as it allows every composition within the gradient to be fabricated in one sample set and easy optimization of the nanocomposite material properties or desired nanostructure without having to create each individual composite target with each individual deposition.

**Figure 2:**
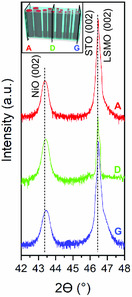
XRD data for LSMO‐NiO nanocomposite thin film Samples A, D, and G.

The LSMO‐NiO nanocomposite thin films were grown in a single deposition on one substrate, as depicted in Figure 1, and cut into smaller individual samples for measurements, making each sample a thin slice. **Figure** **2** shows the XRD data at three different compositions as marked in the key. Sample A has the highest percentage of NiO and Sample G has the highest percentage of LSMO. Sample D represents the composition in the center of the deposited thin film materials. The diffraction data confirms the NiO and LSMO phases were successfully deposited and did not intermix. Reciprocal space mapping was performed and did not discover a clear strain change due to the composition ratio of the two phases (Figure S1, Supporting Information).

**Figure 3:**
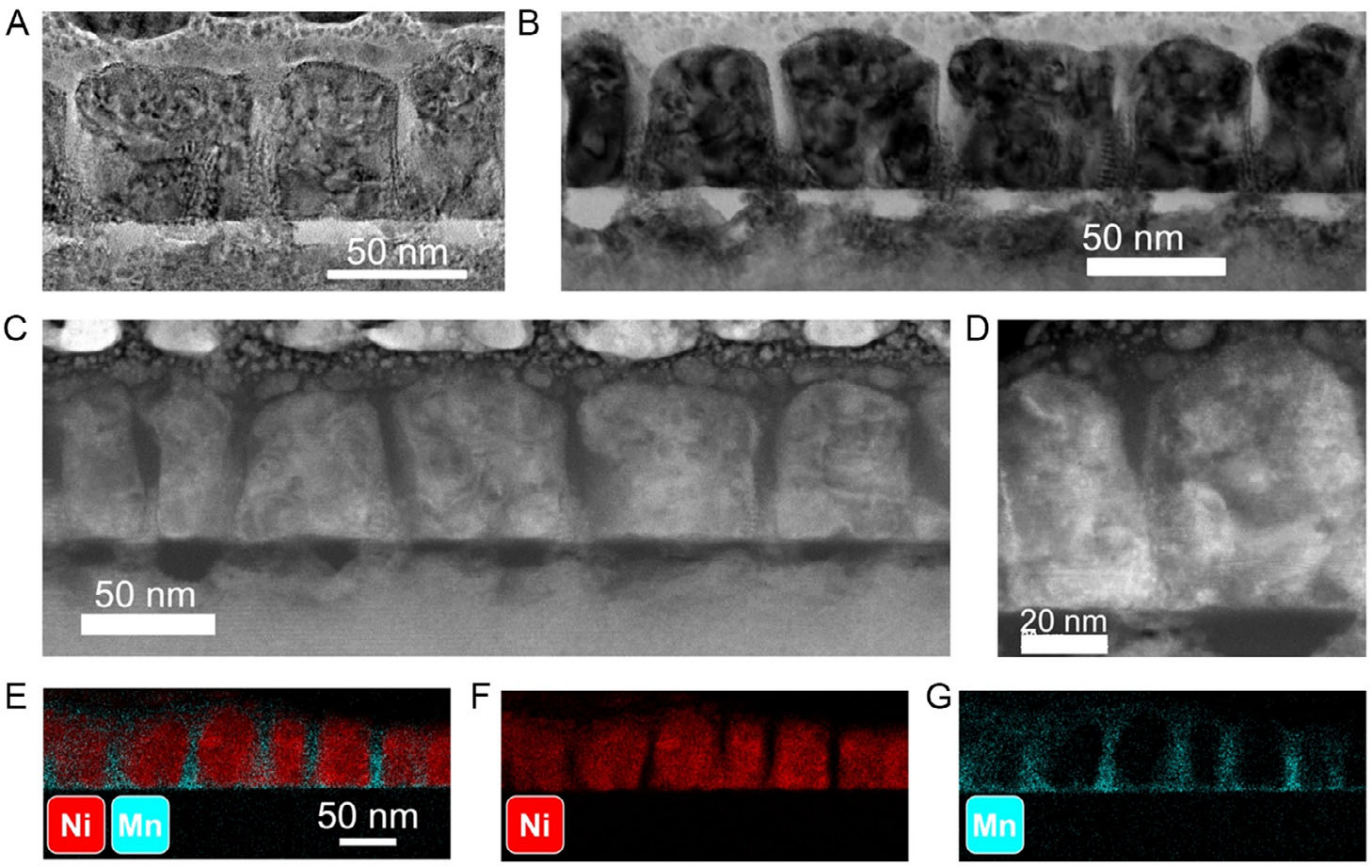
Sample A. A,B)TEM, C,D) STEM, and E) EDX of Ni and Mn combined, F) EDX of Ni, and G) EDX of Mn.

**Figure 4:**
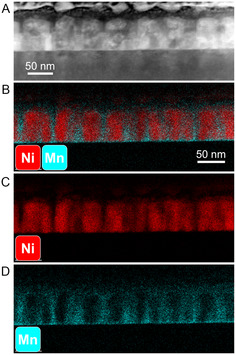
Sample D. A) STEM, B) EDX of Ni and Mn combined, C) EDX of Ni, and D) EDX of Mn.

**Figure 5:**
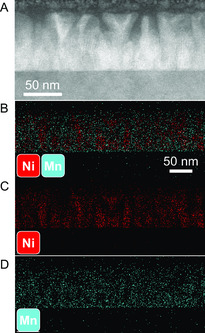
Sample G. A,B) STEM, C) EDX of Ni and Mn combined, D) EDX of Ni, and E) EDX of Mn.

The TEM, STEM, and EDX images for Sample A are shown in **Figure** **3**. The LSMO phase forms the matrix (≈12 nm spacing between nanopillars) in the VAN structure and the NiO phase forms the nanopillars (≈43 nm diameter). The EDX mapping of Ni and Mn confirms the clear phase separation of LSMO and NiO in the system suggesting the effectiveness of the combinatorial method for the VAN growth. **Figure** **4** shows the STEM and EDX results of Sample D, the sample in the middle of the nanocomposite composition gradient. The nanostructure again shows a clear VAN structure, and the diameter of the NiO nanopillars reduces to ≈27 nm with ≈14 nm in spacing. Note that Sample D appears to be NiO‐rich despite the equal amount of laser pulses for the LSMO and NiO targets applied. One possible explanation for this discrepancy could be the differential growth rates of LSMO and NiO. Even if the deposition times for both materials were kept equal, the inherent difference in growth rates between the two materials might result in different compositions. In this case, it is possible that NiO has a faster growth rate compared to LSMO. Thus, for equal deposition time, NiO could constitute a larger proportion of the thin film, making the film appear as NiO‐rich. **Figure** **5** shows the STEM and EDX data for Sample G. The NiO nanopillars in the LSMO matrix become much smaller in diameter (≈14 nm) with ≈17 nm in spacing. In short, the compositional ratio between LSMO and NiO is varied, which directly influences the nanostructure of the nanocomposite system. Specifically, when the composition is altered, the size of the NiO nanopillars is changed. This behavior underscores the role of compositional variation in the control of nanostructure within VAN thin films. In addition to the EDX of the samples done with TEM, a SEM EDX scan was made of a larger area of the sample to measure the amounts of each element present. Table S1 (Supporting Information) shows the atomic percentage of La and Ni present at each of Samples A, D, and G from the SEM EDX image. Performing EDX on both with TEM and SEM images allows for both large‐scale and small‐scale analysis of the samples. The atomic percentage allowed for a calculation of LSMO:NiO ratios for Samples A, D, and G. The LSMO:NiO ratio of A, D, G samples is determined to be 1:3.5, 1:2.7, and 1:1.6, respectively, and this was confirmed with the TEM EDX data. The information confirms the compositional gradient. However, there is a potential error due to overlapping peaks in the SEM EDX spectrum (Figure S2, Supporting Information).

**Figure 6:**
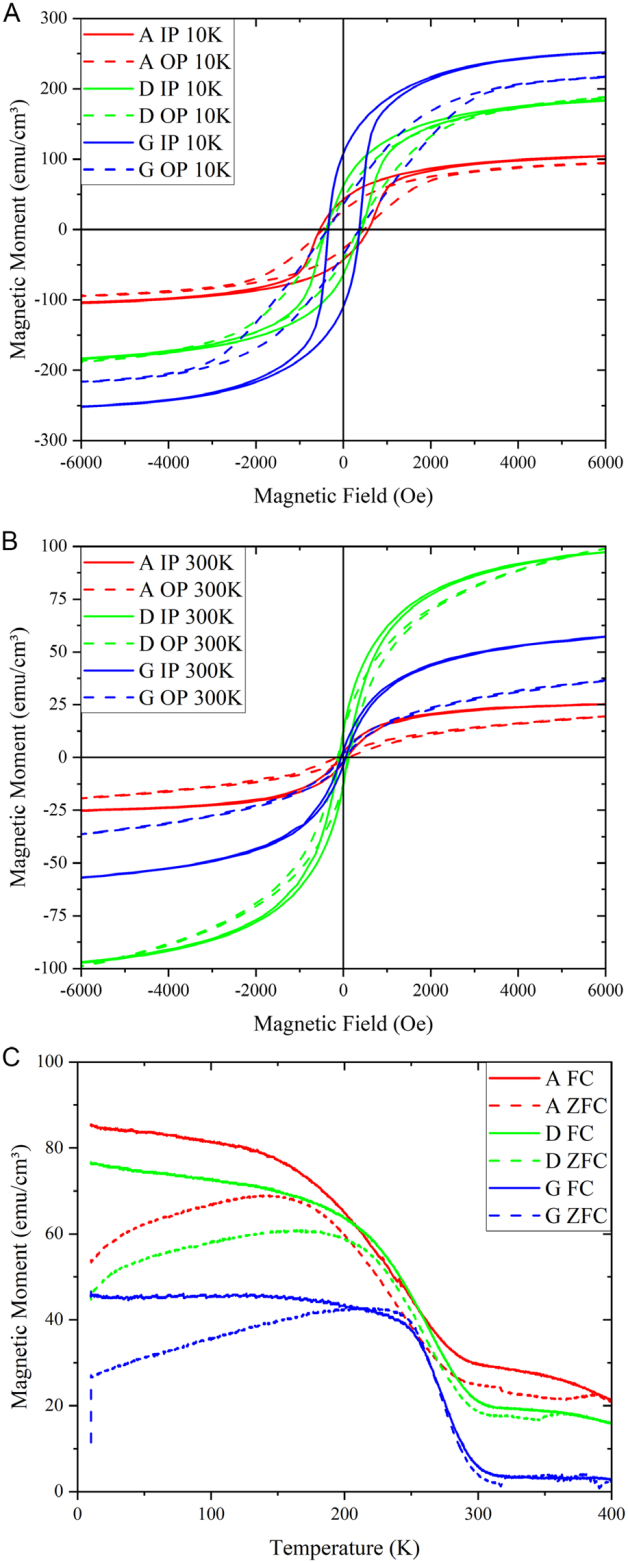
A,B) Magnetization vs magnetic field at 10 K (A) and at 300 K (B). C) OP magnetization versus temperature with an applied field of 1 T.

In terms of the properties of each film, the magnetization versus magnetic field was measured for Samples A, D, and G. **Figure** **6**A,B shows the hysteresis loops for each sample in the in‐plane (IP) and out‐of‐plane (OP) directions at 10 and 300 K, respectively. **Table** **1** shows the magnetic saturation, coercivity, and exchange bias for Samples A, D, and G for both low temperature and room temperature in the IP and OP directions. It is clear that, as the percentage of LSMO increases, the magnetic saturation and magnetic anisotropy both increase due to the increase in the percentage of ferromagnetic LSMO. This can be attributed to the fact that the magnetic data was normalized based on the total film thickness, not specifically on the LSMO composition. As a result, an increase in the LSMO composition would lead to an increase in the overall magnetic saturation of the film as LSMO is the ferromagnetic component. When LSMO's proportion increases relative to NiO, a greater portion of the total film thickness contributes to magnetization, hence, the observed increase in magnetic saturation. The magnetic saturation of the IP direction is also larger than the OP direction for every sample. Except for Sample G, the coercivity is also higher in the IP direction than in the OP direction. A similar trend exists at room temperature as the magnetic anisotropy increases with LSMO and the magnetic saturation increases with increased LSMO percentage except for Sample D, which has a higher room‐temperature magnetic saturation than that of Sample G. The antiferromagnetic NiO could contribute to this as NiO can become ferromagnetic when the features are on the nanoscale, as the spin can become restricted by the dimensions of the material.^[^ ^31^ ^]^ It is believed that the NiO phase in sample D holds the ideal size to be ferromagnetic and thus show the highest magnetic saturation compared to sample A and G, with either too large or too small NiO nanopillars, respectively. At room temperature, the samples all have larger magnetic coercivity in the OP direction and the magnetic coercivity decreases with increased LSMO percentage, suggesting OP anisotropy in magnetic properties for all three compositions. The lower coercivity in the IP direction compared to the OP direction in sample G can be attributed to this matrix‐dominated magnetic behavior. In the LSMO‐NiO nanocomposite, the LSMO matrix forms a continuous phase that promotes magnetic domain alignment along the IP direction. This IP alignment of magnetic domains can reduce the coercivity in this direction, as it is easier for the magnetic domains to flip in response to an external magnetic field. Conversely, the out‐of‐plane direction faces more resistance to domain rotation due to the discontinuity of the LSMO phase in the vertical direction, and effective vertical interface coupling between LSMO and NiO phases. This results in a higher coercivity in the OP direction. Next, the exchange bias results can be analyzed considering the ferromagnetic LSMO and antiferromagnetic NiO in the system. Compared to the coercivity of the samples, there was only a small amount of exchange bias present. The samples measured at room temperature had less results than those measured at 10 K. This can be due to their narrow hysteresis loop. The harder 10 K results developed more exchange bias due to the magnetic spins not being as affected by the thermal properties.

**Table 1:** Magnetic data for Samples A, D, and G for both temperatures and sample orientations

	Sample A				Sample D				Sample G			
	10 K		300 K		10 K		300 K		10 K		300 K	
	IP	OP	IP	OP	IP	OP	IP	OP	IP	OP	IP	OP
Coercivity [Oe]	571	476	82	138	415	339	100	131	366	369	55	56
Saturation [emu cm^−3^]	104	95	26	20	183	188	97	99	251	217	57	36
Exchange bias [Oe]	14	7	0	−3	7.5	−12.5	2	2	11	3.5	2.5	0

Figure 6C shows the OP magnetization versus the temperature for all samples. The Curie temperature of LSMO is 355 K.^[^ ^26^ ^]^ The Curie temperatures of these LSMO‐NiO nanocomposite thin films are lower than that of LSMO with Samples A, D, and G measuring Curie temperatures of 294, 301, and 307 K, respectively. The temperature at which the field cooling (FC) line uses an applied magnetic field to achieve the ideal magnetic saturation of a single‐domain material. The zero‐field cooling (ZFC) line represents the non‐equilibrium effects on magnetization. Where the ZFC line splits from the FC data indicates the temperature at which magnetic domains can form in directions other than that of the idealized single‐domain and reduces the magnetization.^[^ ^32^ ^]^ The bifurcation temperature for Samples A, D, and G are 157, 199, and 250 K, respectively. Lower LSMO percentages reduce the transition temperatures of the nanocomposite materials, and the Curie temperatures and bifurcation temperatures follow the same trend.

**Figure 7:**
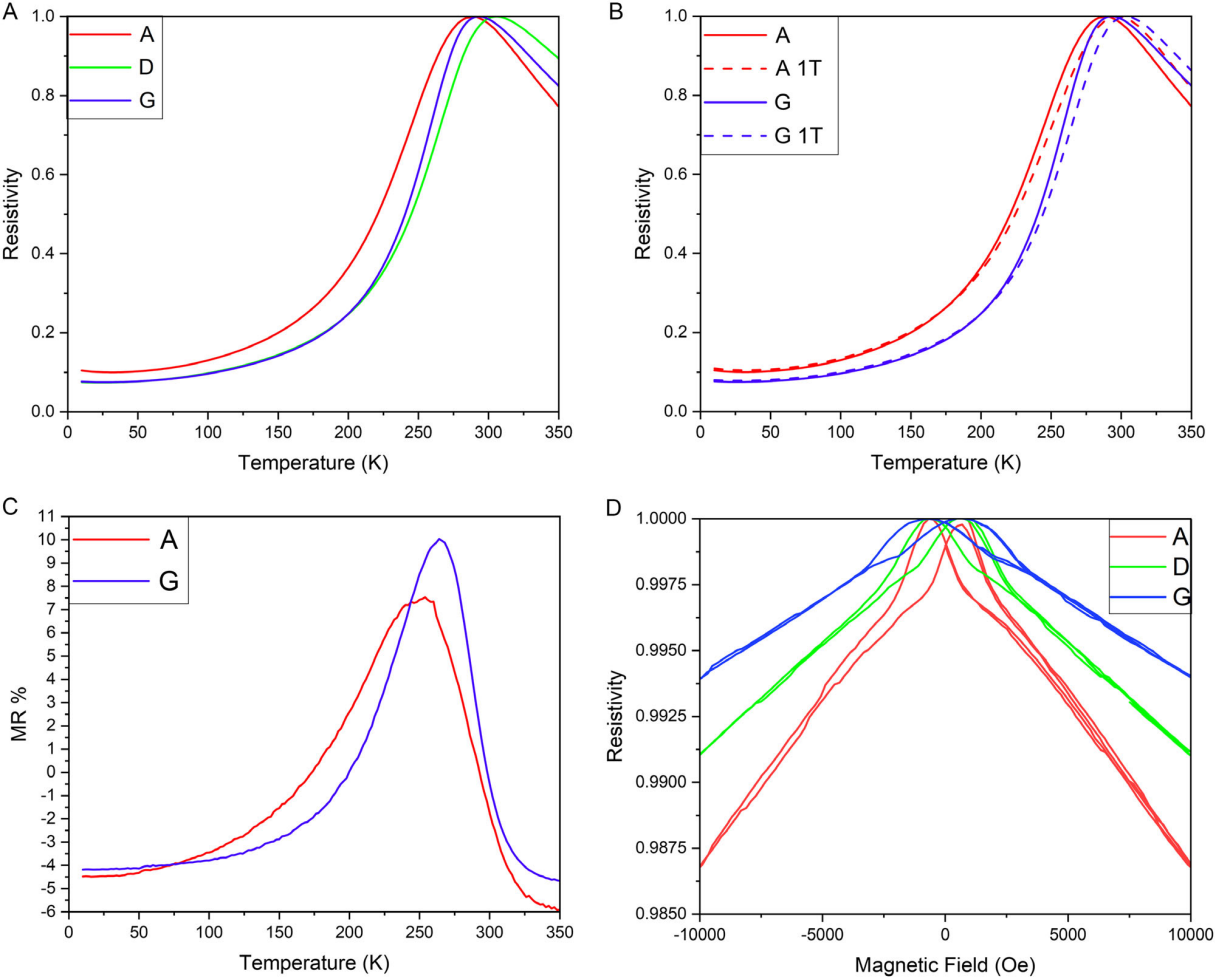
A) Electrical transport, B) electrical transport data with field, C) MR vs temperature, and D) resistivity vs magnetic field.

In addition to magnetic measurements, the electrical transport measurements under 0 and 1 T were conducted to explore the low‐field MR properties of each sample. **Figure** **7**A shows the electrical transport normalized for easier comparison. The temperature where the peak resistivity occurs ranges from 290 K to 306 K. Figure 7B shows the resistivity versus temperature at 0 T and an applied magnetic field of 1 T for the samples at the ends of the composition gradient (A and G). The change in the MR% is calculated in Figure 7C. The MR of Sample A is generally higher than that of Sample G until Sample A hits its peak, from there Sample G has a higher MR. The MR property was also confirmed through resistivity versus magnetic field measurements (Figure 7D), where the trend in the butterfly loops shows that increasing the NiO content increases the MR properties. Sample G (least NiO) has the smallest resistance reduction and lowest MR while sample A (least NiO) has the largest resistance reduction and highest MR. Sample D lies between the two and is more balanced between the two materials, and as NiO concentration decreases, LSMO concentration increases. The change in MR properties is attributed to the NiO secondary phase distribution in the LSMO matrix as NiO is a semiconductor with higher resistivity than that of LSMO. As the NiO composition increases, the transport path in LSMO is effectively blocked, or hindered, by NiO, resulting in higher MR values. Under the applied magnetic field, neighboring spins are effectively aligned and electron tunneling reduces the overall resistivity of the LSMO‐NiO VAN system leading to enhanced MR properties. Therefore, Sample A (NiO‐rich) shows the highest MR% among all the compositions.

The magnetic and electrical transport results suggest that the MR properties, the temperature at which the resistivity peaks, the magnetization bifurcation temperature, and the Curie temperature are tunable based on the composition ratio between LSMO‐NiO. The combinatorial method effectively produced a range of compositions of the LSMO‐NiO VAN system for composition exploration, morphology tuning, and property tuning. This method can be used in many other oxide–oxide VAN systems with two phases that are thermally stable and immiscible. Additionally, this method can also be used for other oxide–metal VAN explorations with a stable metal phase. Future work shall include an additional growth mechanism study into VANs prepared by the combinatorial method compared with the direct growth method since a postannealing step is involved. The pillar diameters are much larger in the combinatorial case (e.g., 50–60 nm to 30 nm) than that of the direct growth (<10 nm in pillar diameters), which is most likely attributed to the annealing process after thin film growth.

## 3 Experimental Section

PLD with a KrF excimer laser (Lambda Physik, *λ* = 248 nm) was used to grow the LSMO‐NiO nanocomposite thin film materials. The films were grown with an energy density of 2.5 J cm^−2^ at 750 °C with a deposition rate of 2 Hz and 200 mTorr O_2_ gas flow. 25 layers of each material were grown on STO (100) substrates. Each layer of LSMO was grown for 10 s. Each layer of NiO was grown for 40 s. After deposition, the samples were annealed for 20 min at 800 °C. The STO substrates were then cut with a diamond saw into smaller (≈3 mm wide) pieces for measurements.

XRD (PANalytical Empyrean diffractometer) determined the crystallography of each sample piece. SEM (Quanta 650) was used for broader EDX analysis. Au contacts were sputtered onto the sample pieces for transport measurements using a physical property measurement system (PPMS) (Quantum Design). The magnet measurements were made using a magnetic property measurement system (MPMS Model 3, Quantum Design). Both IP and OP directions were measured in the magnetization versus the magnetic field. The OP magnetization was measured for the magnetization versus temperature with an applied field of 1 to 1.5 T. Because the samples were small strips, the samples were glued onto a larger STO substrate using GE Varnish to properly fit into the MPMS during the measurements. A scanning electron microscope (FEI Helios Nanolab 600 Dual‐beam) focused ion beam (FIB) was used to cut out precise TEM (FEI Titan 80–300 and FEI Talos F200x) samples for TEM, STEM, and EDX analysis.

## 4 Conclusion

The combinatorial growth method has successfully demonstrated the feasibility of creating a composition gradient of LSMO‐NiO VAN thin films in one specimen as evidenced by the XRD, SEM, and TEM data. In addition to the study of the composition along the gradient, the magnetic and electrical transport properties were measured and compared. Through tuning the composition ratio between LSMO and NiO, the nanostructure of the nanocomposite system was altered from the large NiO pillars (≈43 nm) in Sample A to small NiO nanopillars (≈14 nm) in Sample G. Combinatorial growth explores a new approach for VAN nanocomposite studies, especially in improving the efficiency of VAN thin film materials research. Future work is needed to explore the feasibility of other oxide–oxide systems and oxide–metal systems along with a comparison study between the VANs prepared by the combinatorial method and the conventional direct growth method.

## Conflict of Interest

The authors declare no conflict of interest.

## Supporting information

Supplementary Material

## Data Availability

The data that support the findings of this study are available in the supplementary material of this article.
