# Supplementary material for: Combinatorial Growth of Vertically Aligned Nanocomposite Thin Films for Accelerated Exploration in Composition Variation
Source: Small Sci. 2023 Sep 27;3(11):2300049. doi: 10.1002/smsc.202300049 (PMC11935946; doi:10.1002/smsc.202300049)
Supplement: Supplementary file 1 — Supplementary Material [file SMSC-3-2300049-s001.pdf]

Combinatorial growth of $La_{0.7}Sr_{0.3}MnO_3$ -NiO to create VAN nanostructures on a compositional gradient

Bethany X. Rutherford,[1,2] Di Zhang,[2] Lizabeth Quigley,[1] James P. Barnard,[1] Bo Yang,[1] Juanjuan Lu,[1] Sundar Kunwar,[2] Hongyi Dou,[1] Jianan Shen,[1] Aiping Chen,[2] Haiyan Wang[1,3*]

[1]School of Materials Engineering, Purdue University, West Lafayette, Indiana 47907, USA

[2] Center for Integrated Technology (CINT), Los Alamos National Laboratory, Los Alamos, New Mexico, 87545, USA

[3] School of Electrical and Computer Engineering, Purdue University, West Lafayette, Indiana 47907, USA

*Corresponding Author: hwang00@purdue.edu

Supplementary Information

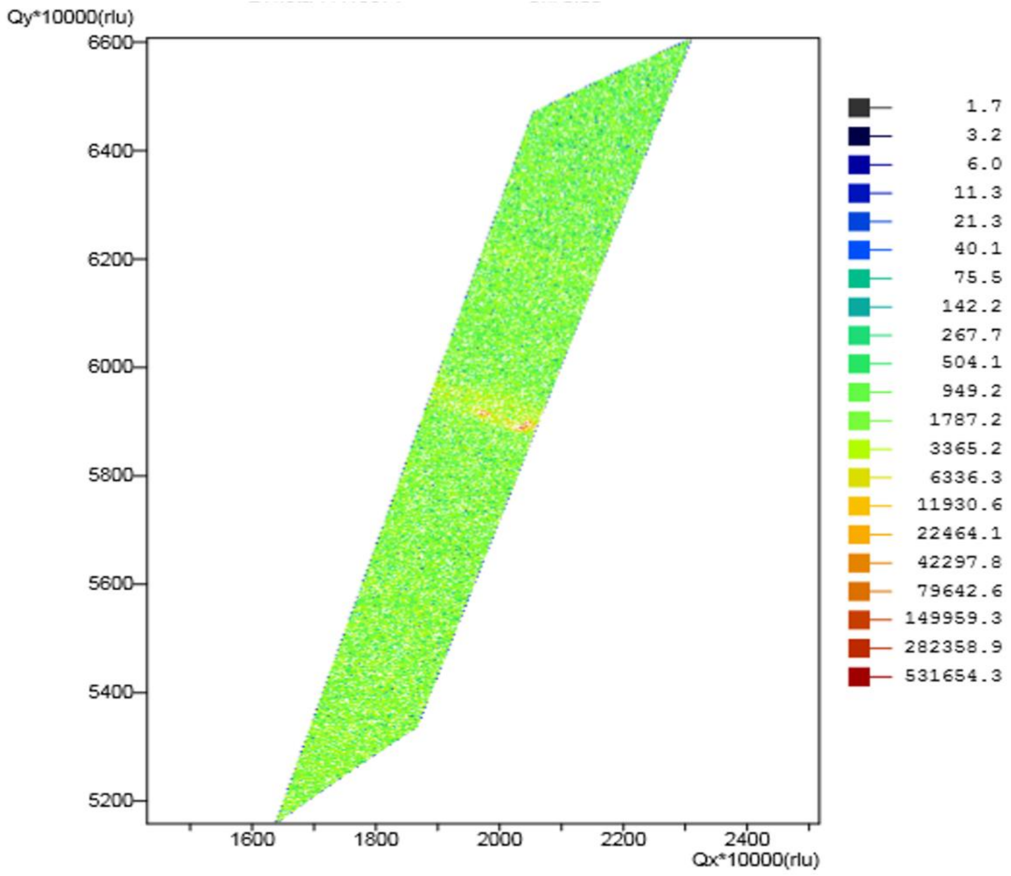

Figure S1. Reciprocal space mapping of the STO 103 peak for Sample G.

Table S1. SEM EDX data for Samples A, D, and G for the at% of Ni and La.

Sample	Ni at%	La at%
A	8.0	1.6
D	6.5	1.7
G	5.6	2.4

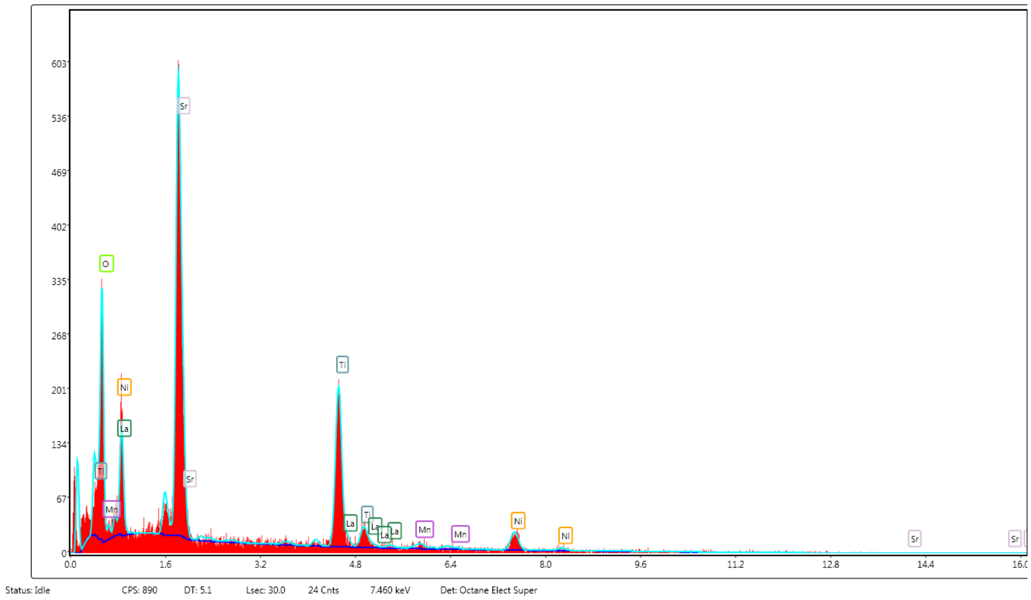

Figure S2. Representative SEM EDX spectrum showing several overlapping peaks.
